# Diabetes-associated neutrophil NETosis: pathogenesis and interventional target of diabetic complications

**DOI:** 10.3389/fendo.2023.1202463

**Published:** 2023-08-03

**Authors:** Yuyan Zhu, Xuan Xia, Qian He, Qing-Ao Xiao, Decheng Wang, Meirong Huang, Xiaolin Zhang

**Affiliations:** ^1^ College of Basic Medical Science, China Three Gorges University, Yichang, China; ^2^ Institute of Infection and Inflammation, China Three Gorges University, Yichang, China; ^3^ Hubei Key Laboratory of Tumor Microenvironment and Immunotherapy, China Three Gorges University, Yichang, China; ^4^ Department of Physiology and Pathophysiology, College of Basic Medical Science, China Three Gorges University, Yichang, China; ^5^ National Clinical Research Center for Metabolic Diseases, Hunan Provincial Key Laboratory of Metabolic Bone Diseases, Department of Metabolism and Endocrinology, The Second Xiangya Hospital of Central South University, Changsha, Hunan, China; ^6^ Department of Interventional Radiology, The First College of Clinical Medical Science, China Three Gorges University, Yichang, Hubei, China; ^7^ Department of Interventional Radiology, Yichang Central People’s Hospital, Yichang, Hubei, China

**Keywords:** NETosis, neutrophil, diabetes mellitus, diabetic complications, inflammation

## Abstract

Neutrophil extracellular traps (NETs) are known as extracellular fibers networks consisting of antimicrobial proteins and decondensated chromatin DNA released by activated neutrophils. NETosis is a NETs-induced neutrophilic cell death which is unique from necrosis or apoptosis. Besides its neutralizing pathogen, NETosis plays a crucial role in diabetes and diabetes-related complications. In patients with diabetes, NETs-releasing products are significantly elevated in blood, and these findings confirm the association of NETosis and diabetic complications, including diabetic wound healing, diabetic retinopathy, and atherosclerosis. This article briefly summarizes the mechanisms of NETosis and discusses its contribution to the pathogenesis of diabetes-related complications and suggests new therapeutic targets by some small molecule compounds.

## 1 Introduction

Neutrophils are identified to be the first-line defense against the invasion of pathogens (such as bacteria, fungi, and viruses) in innate immunity (1). Neutrophils clear away microbes by three mechanisms: (1) release of antimicrobial peptides from neutrophil granules via degranulation; (2) phagocytosis and degradation of bacteria in lysosomes by producing reactive oxygen species (ROS) (2); (3) release of neutrophil extracellular traps (NETs) that allows for the capture of microbes (3). Activated neutrophils immobilize and attack microorganisms and activate other immune cells by releasing depolymerized chromatin from the cell to form a reticulum composed of chromatin-bound to granule peptides named NETs. The formation of NETs represents a form of programmed cell death which is termed NETosis. NETosis is firstly described as cell death distinct from necrosis or apoptosis by Takei et al. in 1996 (4) and termed in 2007 by Steinberg BE et al. (5). Later studies find that NETs destroy and eradicate bacteria, fungi, and viruses in addition to preventing pathogens from migrating outward (3).

Recently, more studies show that NETs perform as double-edged sword. On one side, NETs could kill pathogenic microorganisms and repress the infection-related inflammation; On the other side, excessive production of NETs or impaired clearance of NETs may have severe impact on the organic damage and be involved in many inflammatory diseases, such as type 1 (T1D) or type 2 diabetes mellitus (T2D), and diabetes-induced complications. Herein, we discuss the mechanism of NETosis and its contribution to the pathogenesis of diabetes-related complications, and propose new therapeutic targets.

## 2 Concept and classification of NETs

The concept of NETs, first proposed by Brinkmann et al. in 2004, is a fibrillary meshwork released into the extracellular compartment by neutrophil stimulation (6). It consists mainly of DNA and histones and also contains antimicrobial proteins such as myeloperoxidase (MPO), neutrophil elastase (NE), and cathepsin G (CG) (7). NETs mainly consist of smooth fibers (DNA backbone) of 15-17nm in diameter and spherical structural domains of about 25nm in diameter (6). Activated platelets, phorbol 12-myristate 13-acetate (PMA), lipopolysaccharide (LPS), and pathogen can stimulate neutrophils to promote the production of NETs (8, 9). The process with which neutrophils form NETs is referred to as NETosis.

According different NETosis inducers or depending on NADPH oxidase (Nicotinamide Adenine Dinucleotide Phosphate oxidase, NOX), NETosis can be classified into two types: (1) NOX-dependent NETosis: PMA(phorbol 12-myristate 13-acetate) and LPS can induce NOX assembly and activation to produce ROS (10). MPO makes hypochlorous acid convert to hydrogen peroxide, which in turn activates enzymes such as NE, MPO, and NE in the nucleus. NE and MPO play a synergistic role to enhance chromatin decondensation while N terminus of the histone is cleaved by NE and released into the extracellular compartment. Therefore, the cleaved histone N-terminus can be used as a biomarker to distinguish between two types of NETosis; (2) NOX-independent NETosis: Ionomycin (calcium ionophore) can cause NETosis without the requirement for NOX activation but it can induce calcium overload (11). Calcium overload in cytoplasm activates Protein-arginine deiminase type-4 (PAD4), which citrullinates arginine of histones. Then the positive charge carried by citrullinated histone will be weakened. Consequently, the affinity of DNA (negative charge carrier) and histone will be reduced. Finally, it promotes chromatin decondensation. Then plasma membrane and nuclear envelope (degraded by NE or peroxidated by MPO) permeabilization will be increased. Cytoskeleton and nuclear lamin meshwork will be disassembled by enzyme. Finally, there is plasma membrane rupture and discharge of intracellular DNA (12).

## 3 Typing and mechanism of NETs

### 3.1 Typing

**Figure 1:**
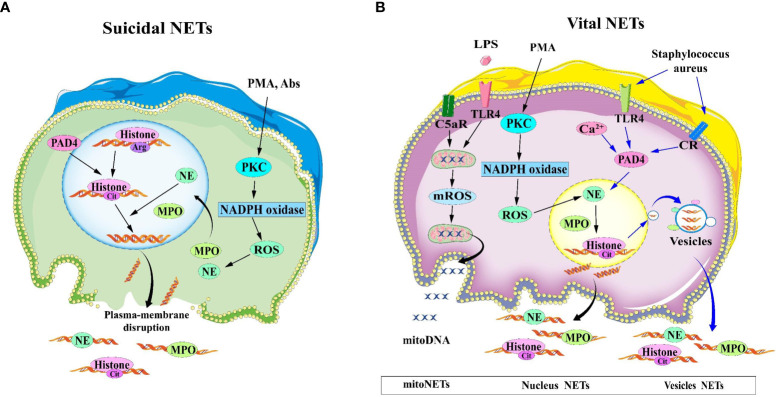
Two mechanisms for the formation of NETs. **(A)** Suicidal NETosis: After neutrophils are stimulated by PMA and Abs, the nuclear and cytoplasmic membranes are disrupted, and the decondensed chromatin is released into the extracellular space, eventually forming NETs. **(B)** Vital NETosis, the nuclear and plasma membranes of vital NETosis remain intact, and chromatin is expelled as vesicles to form NETs. In the case of LPS or complement factor 5a stimulation of neutrophils, mitoDNA can also be released into the extracellular compartment in a ROS-dependent manner. Abs, antibodies; LPS, lipopolysaccharide; NET, Neutrophil extracellular traps; NETosis, neutrophil extracellular trap network apoptosis; PMA, phorbol 12-myristate 13-acetate; ROS, reactive oxygen species.

According to whether the release of NETs is accompanied by neutrophil death or not, NETosis is classified into two categories: suicidal NETosis and vital NETosis (13). The graphical explanation of two types of NETosis are shown in **Figure 1**.

#### 3.1.1 Suicidal NETosis

Suicidal NETosis is the most typical mode of NETs release, lasting for 2-4 hours (14) and the release of NETs to the extracellular compartment is accompanied by neutrophil death. Neutrophils are activated by phorbol 12-myristate 13-acetate (PMA) or extracellular microbes (e.g. *Staphilococcus aureus*) through binding to TLRs, IgG-Fc, compliment, or cytokine receptors, causing the endoplasmic reticulum to release calcium into the cytoplasm, leading to calcium overload (15). Gp91phox phosphorylation results from calcium overload, orchestrating the protein kinase C(PKC) activation and Raf-MEK-ERK pathways, which in turn activates NOX and led to overproduction of ROS (16, 17). In addition, ROS acts as second messengers that ignited MPO and NE to translocate into the nucleus (18). NE hydrolyzes histones and disrupts chromatin packaging, whereas MPO catalyzes chloride oxidation into hydrochloric acid in the presence of hydrogen peroxide. Then MPO disintegrates chromatin and enhances chromatin decondensation with NE. The nuclear membrane (including lamin) is mainly degraded by serine protease (NE and/or PR3) and membrane phospholipid is peroxided by MPO, resulting in gradual separation and decomposition of the nuclear membrane. The decondensed chromatin and proteolytic enzyme is released into the cytoplasm and subsequently into the extracellular space via plasma membrane rupture. So this process induces programmed cell death which is different from apoptosis that requires activation of caspases (19, 20).

#### 3.1.2 Vital NETosis

Compared to Suicidal NETosis, vital NETosis is a more rapid response to *Staphilococcus*. *aureus* (14) and *Candida albicans (* 21) occurring within 5~60 minutes. This vital NETosis occurs without the loss of nuclear or plasma membrane (13) independent of ROS production or Raf/MERK/ERK pathways. Upon recognition of toll-like receptor (TLR) and C3 protein complement receptors on the neutrophil cell membrane, the nuclear membrane bursts, and the nuclear DNA is released as a vesicle wrapped in a budding manner. Simultaneously, different cytoplasmic particles gathered around the vesicles and are expelled, while the inner membrane of the cell remains intact and the activated neutrophils do not die and are still able to maintain their chemotactic and bacterial phagocytic functions (22).

In addition, Yousefi et al. (23) identifies the second type of vital NETosis, a phenomenon that occurs in a shorter time (15min). It is dependent on ROS generation and release of mitochondrial DNA rather than nuclear DNA. it is stimulated by granulocyte/macrophage colony-stimulating factor (GM-CSF) and subsequent short-term stimulation by Toll-like receptor 4 (TLR4) or complement factor 5a (C5a) receptor. The NOX inhibitor diphenyleneiodonium(DPI) results in a complete blockage of mitochondrial DNA release and vital NETosis of neutrophil. There is a unclarified mechanism why the release of mitochondrial DNA from neutrophils and the formation of NETs are not accompanied by neutrophil death.

### 3.2 NETosis testing indicators

Due to the complexity of the structure of NETs, there is no gold markers to ascertain the occurrence of NETosis, combined multiple indicators can help to validate the NETosis. The presence is currently determined mainly by detecting the expression of the following components jointly. (1) circulating double-stranded DNA (Cell-free dsDNA) or DNA-histone complex released from neutrophils is the main component of NETs and is used to detect NETosis. The limitation of this assay is to incapability to distinguished its dsDNA from the small amount of cell-free DNA produced by macrophages (23–25); (2) PAD4-induced histone translation is a vital step in NETosis, and the presence of citrullinated histones 3 (CITH3) identified by immunostaining endorses NETs formation *in vitro* and *in vivo*; therefore, CITH3 or CITH4 level can be considered a marker for the production of NETs and can be widely used for the detection of NETosis (26, 27) (3) Co-localization complexes of extracellular DNA (DNA-NE, DNA-MPO) and neutrophil-derived proteins are highly specific and can be used to determine NETosis (27, 28). Summary on testing indicators of NETosis are seen in **Supplementary Table 1** (29–34).

## 4 NETs and diabetes

### 4.1 Clinical correlation between NETosis and diabetes

Diabetes Mellitus (DM) refers to a metabolic disorder with chronic hyperglycemia. Delayed wound healing and DR are common complications. In contrast to 5mM glucose and 25mM mannitol, Menegazzo et al. (35) discovers that high glucose (25mM) increased the incidence of NETosis. Plasma elastase, mono- and oligonucleotides, and dsDNA levels are higher in T2D patients compared to non-diabetic controls. Mono- and oligonucleotides positively corresponded with glycated hemoglobin A_1c_ (HbA_1c_), while dsDNA is linked to nephropathy and cardiovascular disease These studies suggested that hyperglycemia can induce the release of circulating biomarkers of NETs, and serum levels of NETs in patients are strongly linked with their concomitant renal and cardiovascular diseases (36). Compared with control, the serum levels of IL-6 and TNFα are higher in T2D group and linked with elevated serum levels of dsDNA (35). Wang et al. (37) shows that circulating levels of PR3 and NE activities are higher in T1D patients than control, especially in patients with disease duration <1 year. Elevated NE and PR3 are strongly linked with an increase in the number and titer of positive β-cell autoantibodies, suggesting increased NETosis in the etiology of pancreatic β-cell autoimmunity. These findings highlights that there is positive correlation between higher circulating NE/PR3 level and T1D.Besides, another study finds that there is higher circulating NE/PR3 level in autoantibody-negative T1D. Paradoxically, other studies noted that NE and PR3 levels are significantly lower in patients within a 3-year duration. Furthermore, this have been reported that neutrophil count is decreased in recent-onset T1D patients (38). This relationship T1D and NE/PR3 still remains unveiled.

There is difficult to justify that the elevated circulating NE/PR3 is caused by NETosis or degranulation of neutrophil activation (37). In general, to test the NETosis in diabetes is combined with many indictors such as MPO, NE, PR3, PAD4, LL37(an antimicrobial cathelicidin) and cell-free DNA-histone by ELISA (24). Comparatively speaking, cell-free DNA-histone, PAD4 is more specific to test NETs than NE/PR3.

### 4.2 Mechanisms of NETs involvement in the development of diabetic complications

#### 4.2.1 Mechanisms of NETs-retarding diabetic wound healing

##### 4.2.1.1 PAD4

**Figure 2:**
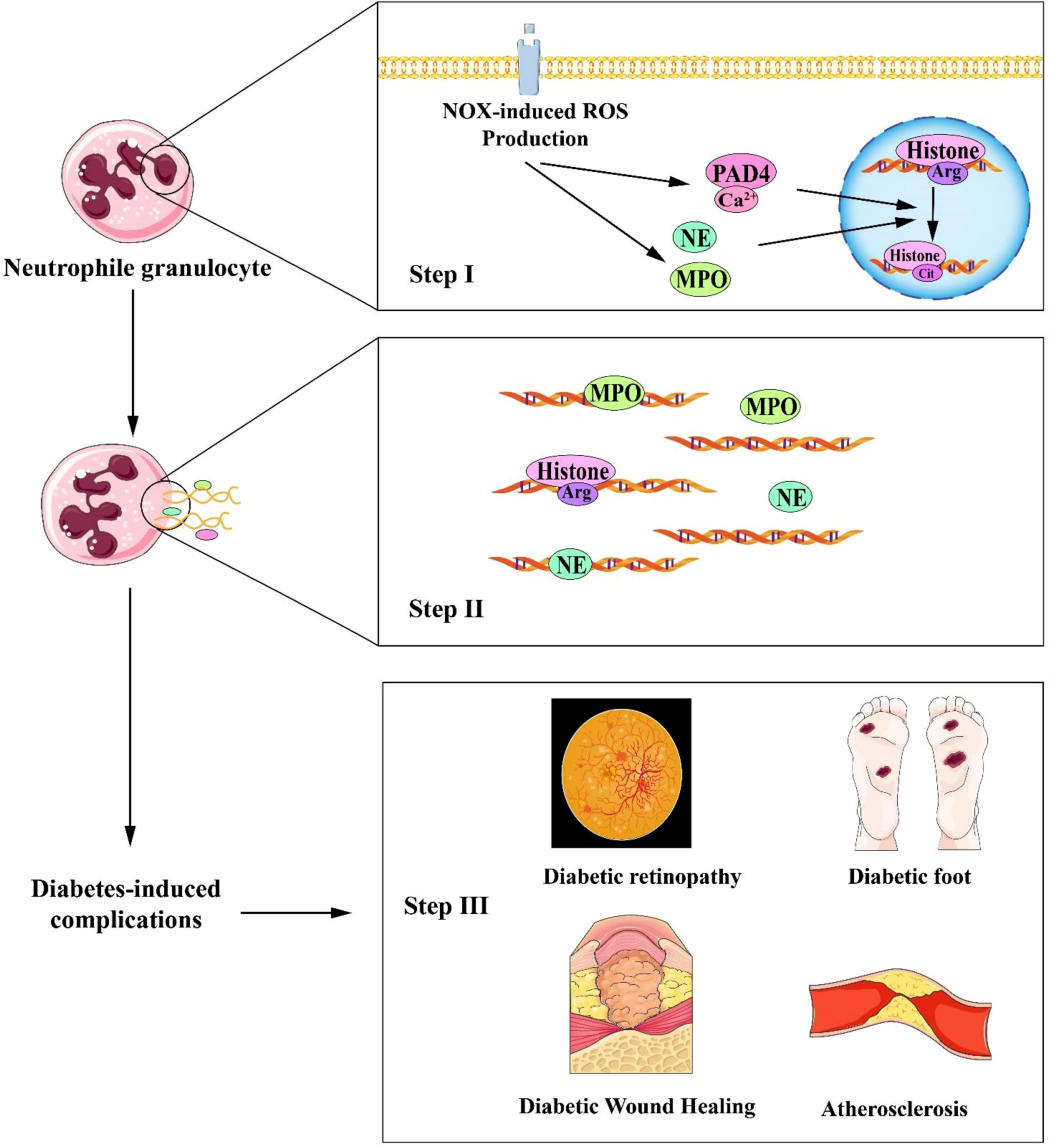
Schematic interpretation of NETosis process in the progression of diabetic complications. STEP I (Top panel): The increase in intracellular calcium ions activates NADPH oxidase (NOX), which stimulates ROS (reactive oxygen species) production. Intracellular calcium ions act as a cofactor for PAD4 (Protein-arginine deiminase type-4), catalyzing histone translation and inducing chromatin decondensation. ROS acts as a second messenger to facilitate the translocation of NE (neutrophil elastase) and MPO(myeloperoxidase.) to the nucleus and promote chromatin decondensation; STEP II (Middle panel): The decondensed chromatin is then released extracellularly along with toxic particles and cytoplasmic proteins; STEP III (Bottom panel): NETs formation eventually leads to diabetes-related complications such as delayed diabetic wound healing, diabetic retinopathy, diabetic foot, and atherosclerosis. NET, Neutrophil extracellular traps; NADPH, Nicotinamide Adenine Dinucleotide Phosphate.

Neutrophil peptidyl arginine deiminase 4 (PAD4) is the sole nuclear PAD that promotes neutrophil histone H3 citrullination (hydrolysis of arginine (Arg) residues to citrulline) and NETosis in skin wounds (7). PAD4 (encoded by *Padi4* in mice) is a calcium-specific enzyme to accelerate NETosis by depolymerizing chromatin. The loss of dense chromatin structure is induced by PAD4-mediated histone hypercitrullination, which lowers affinity between DNA and histone and separation of histones from DNA (20, 39) (See in **Figure 2**).

Wong et al. shows that PAD4 expression in neutrophils is significantly elevated and NETs release is increased in diabetes compared to healthy controls (40). Also, Wong et al (40) shows that PAD4 expression is upregulated 4-fold in neutrophils from diabetic patients compared with healthy controls. In comparison to normoglycemic mice, there is more CITH3 expression in diabetic mice’s wounds. In excisional skin lesions, wild-type (WT) mice developed a significant amount of NETs, but *Padi4* ^-/-^ mice shows no NETs and healed faster than WT control. In *Padi4-/-* mice, re-epithelialization occurred three times faster than in WT mice. In diabetic *Padi4^-/-^* mice, there is no delayed wound healing, showing that hyperglycemia triggers neutrophils to overproduce PAD4 and NETs, revealing NETs as a deterrent component in slowing down wound healing.

Inhibition of PAD4 by chloramine reduces reticulocytes and promotes wound healing in diabetic mice. In diabetic and WT mice, DNase I, an enzyme in the degradation of NETs, expedite wound healing. In contrast to increased CITH3 signaling in WT mice, no CITH3 alteration is found in *Padi4* ^-/-^ mice and wound healing is accelerated. Thus, DNase I and PAD4 inhibition may be applied as wound-healing treatments (40).

##### 4.2.1.2 Neutrophil elastase (NE)

NE is a serine proteinase and lysozyme stored in azurophilic granules in neutrophils (41, 42) and is a key component in the formation of NETs that causes wound matrix to degrade and delays wound recovery. Wang et al. (37) measures the dynamic changes of NE/PR3(neutrophil granule proteinase 3) in NOD(non-obese diabetic) mice. Circulating NE/PR3 enzyme activity is significantly higher in diabetic mice than onset stage of diabetes. Then, NE/PR3 activity in diabetic mice eventually drop to baseline, probably because of the destruction of pancreatic β-cells leading to the termination. neutrophil elastase. NE/PR3 enzyme activity is higher in deteriorating wounds than in healing wounds. Compared to unhealed wounds, serum NE level is decreased in patients with fully healed wounds and tended to decrease in patients with spontaneous healing. NE can promote the NETosis through the Gasdermin D (GSDMD) pores (43). Once gasdermin D cleavage into its p30 pore-forming fragment, these pores allow the entry of enzymes(such as caspase-11, NE) into the nucleus, promoting histone citrullination and chromosome depolymerization (44, 45) of the NE pathway. Guamerin is a novel leukocyte elastase inhibitor. Lee et al. (46) explores the effects of guamerin on exposed tongue-prick wounds and finds that recombinant elastase inhibitor guamerin (Reig) resulted in faster wound healing, significant recovery of re-epithelialization, and faster inflammation in skin wounds regression is accelerated. Therefore, elastase inhibitor protects and prolongs the stability of temporary matrix fibrin at the wound site, thus enhancing the process of tissue repair.

##### 4.2.1.3 MPO

MPO is a major particle-resident protein associated with chromatin densification during NETs disease. MPO catalyzes chloride oxidation into hydrochloric acid in the presence of hydrogen peroxide. Neutrophils from patients with MPO deficiency do not release NETs when stimulated by PMA, *staphylococcus aureus*, or *group B streptococci*. MPO inhibitor only delays NETosis, indicating that MPO inactivation is valuable in treating NETosis (47). Although MPO does not directly decondense chromatin in isolated cell nuclei, nor does it degrade histones *in vitro*, it can help NE to promote chromatin densification (18). How MPO facilitate chromatin decondensation or it mediate oxidation of DNA is still unknown.

##### 4.2.1.4 Protein kinase C (PKC)

Activation of protein kinase C—especially the beta (PKC-β) and delta (PKC-δ) can result in diabetic vascular complications. In addition to PAD4 for NETosis, PKC(especially PKC-β) is considered to be one of the key players in the induction of NETosis (48, 49). Higher levels of PKC-βII is observed in NETosis in the blood of patients with diabetic foot ulcers (DFUs). Ruboxistaurin is a specific PKC βII inhibitor that promotes angiogenesis while also blocks neutrophil proliferation of granulocytes and alleviates diabetic foot ulcers (26).

#### 4.2.2 Diabetic retinopathy

There are inflammatory cell infiltration and their adherence to vascular endothelium as features of diabetic retinopathy (DR). NETs are significantly elevated in the serum of patients with proliferative diabetic retinopathy (PDR), while patients with DR have higher levels of circulating NETosis biomarkers (NE,MPO) (50, 51). Wang et al. (51) verifies that retinal neutrophil infiltration in the retina accelerates leukocyte sludge in the retina, neutrophil-endothelium adhesion, and inflammatory responses. By co-labeling of CITH3 and MPO, typical NETs is observed in the vitreous and retina in patients with diabetes, showing decompressed DNA structures and co-localized proteins, suggesting that NETs formation takes a part in the pathogenesis of DR.

Meanwhile, anti-VEGF treatment reduces the formation of NETs in DR (52). Binet et al. (53) discovers that vascular remodeling in retinal lesions is linked to delayed neutrophil recruitment, which result in cellular senescence. After that, the senescent vasculature secretes cytokines and other substances that induce chemotaxis of neutrophils and causes NETosis. Neutrophils promotes pathological apoptosis of senescent vessels by expelling NETs and eliminating pathologic senescent vessels. These findings suggest that neutrophils clear away pathologic senescent vessels by releasing NETs, thereby preparing the ischemic retina for reparative vascular regeneration (54).

#### 4.2.3 Atherosclerosis

Atherosclerosis (AS) is a low-grade inflammatory disorder of the vascular wall suffered from dyslipidemia. NETs promote atherogenesis and are thought to directly induce endothelial dysfunction (as initiation stage of atherosclerosis) by activating endothelial cells (25). NETs formation is induced in apolipoprotein E (ApoE)-deficient(*ApoE* ^-/-^) mice after 8-week high-fat diet-induction. A powerful IL-1/Th17 response facilitates atherogenesis which is brought on by NETs-mediated macrophage initiation (25). Cholesterol crystals can induce NETs-associated with atherosclerosis. Cholesterol crystals can bind to CD36 on macrophages and then initiate IL-1β production by macrophages, leading to activation of the Th17 response and further recruitment of immune cells into atherosclerotic lesions (55–58). Warnatsch et al. finds that *ApoE* ^-/-^ mice lacking NE and PR3 have reduced atherosclerotic lesions and fewer NETs than *ApoE* ^-/-^ control (55, 59). In addition, oxidized low-density lipoprotein (ox-LDL) as an atherosclerosis-inducer (60), can also induce NETs through ROS formation in a concentration- and time-dependent manner. Ox-LDL-induced NETs may be mediated by its simultaneous binding to neutrophilic TLR-2 and TLR-6 (56). In **Figure 2**, the steps of NETs interpret the progression of diabetic complications.

## 5 Small molecule compounds to inhibit NETosis in diabetes

Recent studies have found certain drugs or chemicals can inhibit NETosis in inflammatory diseases (e.g. diabetes). The first-line drug for the treatment of type 2 diabetes, metformin has intrinsic activity to inhibit cellular and biochemical processes that result in NETosis (60). The PKC-βII isoform is considered a mediator of the chronic complications of hyperglycemia (61), and Menegazzo et al. (62) shows that metformin inhibits the PKC-NADPH oxidase pathway, preventing membrane translocation of PKC-βII and inhibiting NADPH oxidase activity in neutrophils, thus reducing NOX-dependent NETosis and resulting in lower concentrations of NET components in the plasma of diabetic patients. In addition, metformin has recently been demonstrated to influence the nuclear pore complex, involving remodeling of the nucleus (63). Ruboxistaurin is a selective inhibitor for PKC βII (26)which has deeply involved in NETs (48). Ruboxistaurin can also decrease the percentages of H3Cit+ cells (histone citrullation-positive neutophil) in both peripheral blood and wound areas. It promotes wound closure and stimulated angiogenesis in diabetic mice by preventing excessive neutrophil’s NETosis. So ruboxistaurin can gain benefit on vessel protection and reduces albuminuria and maintained eGFR(Estimated Glomerular Filtration Rate) over 1 year in persons with type 2 diabetes and nephropathy (64). Ruboxistaurin can further gain mild beneficial effects on visual loss in clinics (65).Hydrogen sulfide (H2S) is an endogenous signaling molecule that attenuates NETosis and accelerates diabetic wound healing by blocking ROS-mediated activation of MAPK ERK1/2 and p38 (66), suggesting that adding H2S precusor and cystathionine γ-Lyase(H2S synthetase) activator might become a future drug target to prime diabetic wound to heal. Other NETosis-related compounds have been investigated in several disease model other than diabetes (see in **Supplementary Table 2**) (67–82).

Although there are many studies on NETosis inhibitors, there are simply a few reports on its application on diabetes or diabetic complications. Whether these candidate inhibitors (including vitamin D) can treat diabetic complications still needs to be verified in double-blinded randomized clinical trial (83). Silybin (complex extract of flavonolignans from Milk thistle) also have been approved as an inhibitor of NETs to treat non-alcoholic steatohepatitis (NASH) which is predisposed to diabetes (84). We supposed that these inhibitors (seen in **Supplementary Table 2**) might be used to treat not only for late stage of diabetes, but also to prevent the diabetogenesis and diabetic progression. This is an interesting and promising direction for research.

## 6 Conclusion and perspectives

This review describes the possible mechanisms of NETosis and highlights its significance in diabetes-related complications including involvement in diabetic wound healing, DR, and the development of atherosclerosis. The development of specific NETosis inhibitors might reduce NETs and decrease the severity of diabetic complications. In conclusion, NETosis is a momentous component of the list of possible therapeutic targets for diabetic complications, and worthy to further investigation in near future.

## Author contributions

YZ and XX drafted the manuscript, retrieved literature, and drew the figures. QH and Q-AX reviewed all the literature and give suggestions on figure in the manuscript. DW revised the manuscript and gave suggestions on the figures in the manuscript. XZ and MH drafted framework of this review, edited partly, contributed substantially by giving key suggestions during the manuscript writing. All authors contributed to the article and approved the submitted version.
