# Supplementary material for: Diabetes-associated neutrophil NETosis: pathogenesis and interventional target of diabetic complications
Source: Front Endocrinol (Lausanne). 2023 Aug 3;14:1202463. doi: 10.3389/fendo.2023.1202463 (PMC10435749; doi:10.3389/fendo.2023.1202463)
Supplement: Supplementary file 1 [file Table_1.docx]

| **Supplementary table 1 Comparison of NETosis markers and their detection methods** | | | | |
| --- | --- | --- | --- | --- |
| **NETs detection component** | **Detection method** | **Advantages** | **Disadvantages** | **References** |
| Cell-free DNA | Fluorescent staining method (often using Picogreen dyes) | Wide range of applications | Cannot be distinguished from DNA derived from other cell death | (1, 2, 25) |
| CitH3 | Flow cytometry | Fast and reliable, excluding bias caused by different observers | Detection of CitH3-negative NETs may be overlooked | (27, 30) |
| NE | Protein blotting | Qualitative and semi-quantitative aspects of NE can be resolved in terms of protein expression | Many variables, time-consuming | (18, 63, 64) |
|  | Microtiter plate color development method | High parallelism and stability with low sample usage | When the sample size is too small, the influence of individual values is obvious |  |
| MPO | ELISA | Low cost, automatable | Poor repetition | (65-67) |
|  | Flow Cytometry | Simple operation and high accuracy | Detection of cells generally detects MPO in the cytoplasm, causing some error in the quantification of NETs |  |
|  | Fluorescence immunochromatography | Low detection limit, fast detection | Poor anti-interference ability |  |
| MPO/NE-DNA | ELISA | Highly-specific | Lack of objectivity and difficulty in quantifying the formation of NETs | (27, 68) |

Abbreviation: NET, Neutrophil extracellular traps; CITH3,citrullinated histones; NE, neutrophil elastase; MPO, myeloperoxidase.

| **Supplementary table 2 Small molecule compounds to inhibit NETosis** | | | | |
| --- | --- | --- | --- | --- |
| **Name** | **Molecular weight** | **Structural formula** | **Mechanism** | **References** |
| Methotrexate | 454.44 | 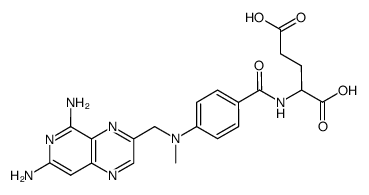 | Reduces ROS formation, indirectly inhibits NET production | (69) |
| Prednisolone | 360.44 | 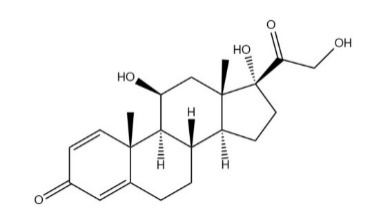 | Inhibits the production of ROS and inflammatory mediators, and reduces the production of NETs | (70, 71) |
| Diphenyleneiodonium (DPI) | 314.55 | 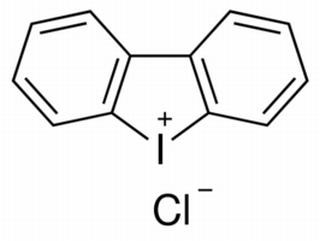 | Inhibition of ROS production and complete blockage of mitochondrial DNA release by neutrophils | (23, 72) |
| Cl-amidine | 310.78 | 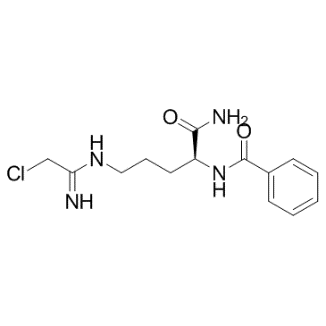 | Inhibition of PAD4 and reduction of NETs | (7, 73, 74) |
| Aminobenzoic acid hydrazide (ABAH) | 151.17 | 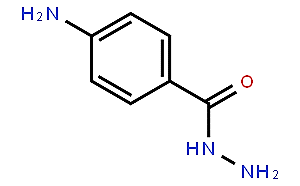 | Inhibits MPO and delays lesions in NETs | (41, 75, 76) |
| Trimethylamine N-oxide (TMAO) | 75.11 | 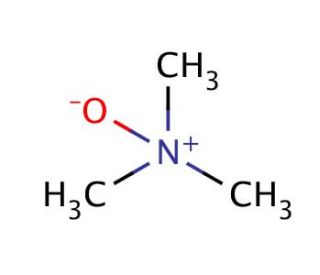 | Inhibition of NADPH oxidase activity and reduction of NETs production | (77) |
| Hydroxychloroquine (HCQ) | 335.87 | 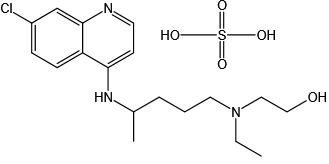 | Inhibition of TLR-4, ROS and IL-2 production expression by PAD9 and Rac8 | (78, 79) |
| PF-1355 | 321.35 | 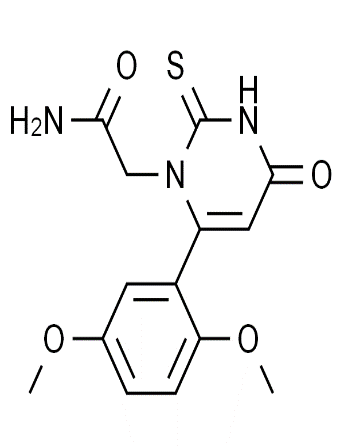 | Inhibits MPO and suppresses the formation of NETs and ICs | (80) |
| AZD9668 | 545.53 | 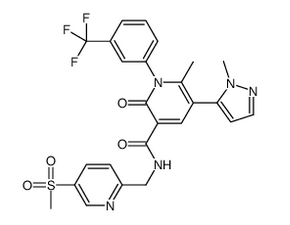 | Inhibits neutrophil elastase/IL-1β IL-6, IL-8, TNFα, and indirectly inhibits NETs production | (81) |
| BMS-P5 | 472.58 | 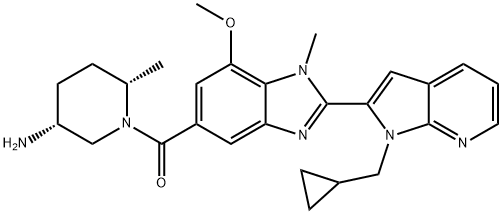 | Inhibits PAD and indirectly inhibits NETs production | (82, 83) |
| Tetrahydroisoquinoline derivatives (THIQs) | 589.17 | 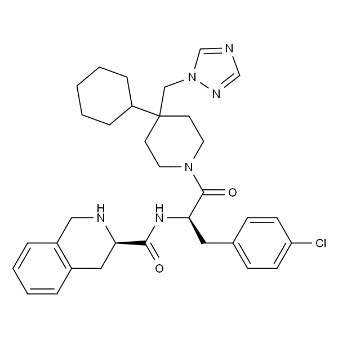 | Inhibiting different stages of NET formation without weakening neutrophil normal functions | (84) |

Abbreviation: NET, Neutrophil extracellular traps; MPO, myeloperoxidase; PAD4, Protein-arginine deiminase type-4; ROS, reactive oxygen species; PKC, protein kinase C; NADPH, Nicotinamide Adenine Dinucleotide Phosphate; IL; interleukin; TNF, tumor necrosis factor; TLR toll-like receptor.
